# Body Temperature Monitoring and SARS Fever Hotline, Taiwan

**DOI:** 10.3201/eid1002.030748

**Published:** 2004-02

**Authors:** S. Cornelia Kaydos-Daniels, Babatunde Olowokure, Hong-Jen Chang, Rachel S. Barwick, Jou-Fang Deng, Ming-Liang Lee, Steve Hsu-Sung Kuo, Ih-Jen Su, Kow-Tong Chen, Susan A. Maloney, the SARS International Field Team

**Affiliations:** * West Virginia Bureau for Public Health, Charleston, West Virginia, USA; † Centers for Disease Control and Prevention, Atlanta, Georgia, USA; ‡ World Health Organization, Geneva, Switzerland; § Health Protection Agency, Regional Surveillance Unit West Midlands, Birmingham, United Kingdom; ¶ Bureau of National Health Insurance, Taiwan; # Taipei Medical Association, Taipei, Taiwan; ** Tzu-Chi University, Hualien, Taiwan; †† Taipei Economic and Cultural Representative Office, Washington, DC, USA; ‡‡ Center for Disease Control, Taiwan

**Keywords:** hotlines, telephone hotlines, severe acute respiratory syndrome, SARS, Taiwan, Asia, fever surveillance, community surveys

## Abstract

In Taiwan, a temperature-monitoring campaign and hotline for severe acute respiratory syndrome (SARS) fever were implemented in June 2003. Among 1,966 calls, fever was recorded in 19% (n = 378); 18 persons at high risk for SARS were identified. In a cross-sectional telephone survey, 95% (n = 1,060) of households knew about the campaign and 7 households reported fever.

Fever is one of the first signs of severe acute respiratory syndrome (SARS) (*1*–*3*). Persons with fevers initially attributed to other illnesses have caused outbreaks of SARS in hospitals and the community (*1*–*7*). This finding highlights the need for early recognition of cases.

On June 1, 2003, in Taiwan, a National Temperature Monitoring Campaign and SARS fever hotline were launched. These were intended to raise public awareness about SARS (and fever as an early sign of SARS), improve early detection of possible SARS cases, and prevent SARS transmission. In the campaign, fever was defined as forehead or axillary temperature >37°C, oral temperature >37.5°C, or tympanic or rectal temperature >38°C (*8*) (See Appendix).

In conjunction with this campaign, persons with fevers were encouraged to call a toll-free SARS fever hotline. The hotline objectives were to appropriately triage persons with fever, reduce clinic visits by the “worried well,” identify persons at high risk for SARS, reduce opportunities for SARS exposure, and increase the public’s sense of security.

Both the body-temperature monitoring campaign and the hotline were publicized through television, posters, fliers, radio, the Internet, magazines, and newspapers. We describe and evaluate the body-temperature monitoring campaign and the SARS fever hotline.

## Methods

Our investigation evaluated the community-wide body-temperature monitoring campaign and SARS fever hotline in the city of Taipei, which makes up 11.8% of the population of Taiwan (population of Taiwan, 22.51 million [*9*]). We analyzed data from three sources: hotline call data reported to the Bureau of National Health Insurance for all of Taiwan; hotline call data for Taipei; and data from a telephone survey of Taipei residents. Data were evaluated for the period June 1–10, 2003, corresponding to the duration of the body-temperature monitoring campaign as well as the first 10 days of the hotline (See Appendix).

### SARS Fever Hotline Data

**Figure 1:**
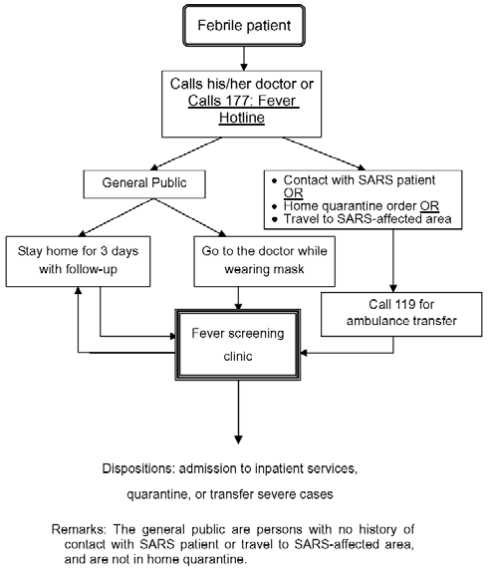
Triage algorithm for febrile patients, severe acute respiratory fever hotline, Taipei, June 2003.

Throughout Taiwan, each local medical association responsible for operating the fever hotline in its city or county provided daily reports to the Bureau of National Health Insurance. The total number of calls and the advice given to the caller were reported. Because operation of the hotline varied by locality, further analysis was limited to Taipei city, where the Taipei Medical Association staffed the hotline and 52 physicians worked 6-hour shifts between 8:00 am and 10:00 pm daily. Physicians were provided with an algorithm (Figure 1) for triaging callers and evaluating SARS risk level. Persons at high risk for SARS were defined as those with fever plus any recent history of quarantine, travel to SARS-affected areas, or contact with SARS cases. Physicians also received a form to document all calls. Data fields on the form were caller or patient name, sex, district of residence, telephone number, a section for comments, a checklist of topics discussed, and diagnosis. The diagnosis field was narrative; therefore, data were classified into broad categories based on the body part or system affected. The hotline data collection forms did not include anatomic site of temperature measurement, therefore, for our evaluation, fever was defined as a recorded body temperature of ≥38°C (See Appendix).

### Cross-Sectional Telephone Survey of Taipei Residents

A telephone survey of Taipei city residents was performed to assess knowledge of the body-temperature monitoring campaign and use of the fever hotline. Households in Taipei were selected for participation in the survey on June 13 to 14, 2003, using a simple random sample of home telephone numbers. Interviewers explained the survey to potential respondents and obtained verbal consent before administering a brief questionnaire.

The Yates corrected chi-square test and the Fisher exact test were used for comparison of groups.

## Results

### Taiwan SARS Fever Hotline Data

**Figure 2:**
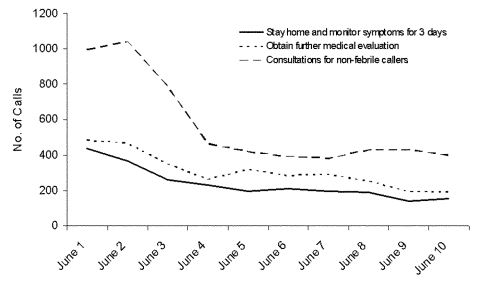
Advice given to callers to severe acute respiratory syndrome fever hotline, Taiwan, June 2003.

During June 1 to 10, a total of 11,228 calls were made to Taiwan’s population-wide fever hotline (Figure 2). Persons were advised to seek further medical evaluation (through family physician, fever clinic, or by ambulance) in 28% (n = 3,100) of calls, and persons were advised to remain at their residence and monitor symptoms in 21% (n = 2,385) of calls. Neither of these recommendations was given in 51% (n = 5,743) of calls.

### Taipei SARS Fever Hotline Data

During June 1 to 10, a total of 1,966 calls were made to the fever hotline in Taipei. Body temperature was recorded for 51% (n = 1,012) of calls. A temperature of ≥38°C (range 34.0–41.0°C, median 37.6°C) was recorded in 37% (n = 378) of calls in which body temperature was recorded. Of the 453 calls with diagnoses, the most common were respiratory and gastrointestinal syndromes (Table 1) for persons with or without fevers. Among calls for which the recommendation given was documented, callers with fever were more likely than callers without fever to be advised to see a physician for further medical evaluation (p < 0.001) or go to a fever clinic (p < 0.001) (Table 2).

**Table 1:** Diagnoses reported for callers by recorded body temperature, Taipei SARS fever hotline, June 1–10, 2003 (n = 1,966)^a^

	Body temperature		
Diagnosis or syndrome	Fever ≥38°C n (%)	No fever n (%)	Unknown/unrecorded
Possible SARS	5 (1.3)	2 (0.3)	11 (1.2)
Respiratory	65 (17.2)	99 (15.6)	40 (4.2)
Dermatologic	0 (0)	1 (0.2)	3 (0.3)
Head-related^b^	6 (1.6)	10 (1.6)	2 (0.2)
Gastrointestinal	21 (5.6)	47 (7.4)	14 (1.5)
Genitourinary	7 (1.9)	8 (1.3)	4 (0.4)
Other	27 (7.1)	31 (4.9)	50 (5.2)
Unknown/missing	247 (65.3)	436 (68.8)	830 (87.0)
Total	378	634	954

^a^SARS, severe acute respiratory syndrome. ^b^Includes neurologic.

**Table 2:** Reported advice given to persons by recorded body temperatures, Taipei SARS fever hotline, June 1–10, 2003 (n = 1,966)^a^

Advice given		Body temperature		
		Fever ≥38°C n (%)	No fever n (%)	Unknown/unrecorded n (%)
Stay home and monitor^b^		19 (5.0)	42 (6.6)	21 (2.2)
See physician^b^		116 (30.7)	55 (8.7)	40 (4.2)
Go to fever clinic		21 (5.6)	2 (0.3)	5 (0.5)
Call ambulance		1 (0.3)	0 (0)	0 (0)
Other		3 (0.8)	10 (1.6)	13 (1.4)
Unknown or unrecorded^b^		221 (58.5)	535 (84.4)	888 (93.1)
Total	378		634	954

^a^SARS, severe acute respiratory syndrome. ^b^Advice given to 18 callers at high risk for SARS: for 5 with fever: see physician (1 caller); unknown or unrecorded (4 callers). For 2 callers with no fever: unknown or unrecorded (2 callers). For 11 callers with unknown body temperature: stay home and monitor (1 caller), unknown or unrecorded (10 callers).

Eighteen (0.9%) persons were identified as being at high risk for SARS. Of these, 5 (28%) had fever, 2 (11%) had no fever, and temperature was unrecorded for 11 (61%). One person with unrecorded temperature was advised to stay home and monitor symptoms, and one person with a fever was advised to visit a physician. The advice given to the remaining 16 persons was not recorded.

### Cross-Sectional Telephone Survey of Taipei Residents

Of the 4,000 telephone numbers dialed, 2,999 numbers were invalid, unanswered, or refusals. Of the 1,111 survey participants, 58% (n = 643) were female, the median age was 47 years (range 20–91), and the median number of people per household was 4 (range 1–17). Ninety-five percent (n = 1,060) and 71% (n = 791) of respondents had heard about the body-temperature monitoring campaign and the fever hotline, respectively. The most common sources of information about the campaign were television (86%), newspapers or magazines (36%), and neighborhood leaders (26%). Twice-daily temperature monitoring of at least one household member was reported by 95% (n = 1,012) of persons who knew of the campaign and 76% of the 51 who were unaware of the campaign (n = 39).

Seven (0.63%) respondents reported a fever in their household during June 1 to 10, 2003. Although five (71%) of these fevers occurred in households in which the respondent knew about the hotline, in only one case was the fever hotline used; actions of the remaining six are unknown. The person who called the hotline reported that the advice given by the physician was to stay home and monitor the symptoms and that the advice was followed. Among all respondents, 24% (n = 267) said that they would call the fever hotline for advice, 54% (n = 605) would go to a hospital, 19% (n = 207) would visit an outpatient clinic, and 1% (n = 10) would do nothing and wait to see if the fever disappeared. The remaining respondents refused or said they would do something else.

## Discussion

The population-wide body-temperature monitoring campaign and fever hotline were innovative interventions aimed at raising public awareness about SARS, improving early detection of fever, and providing appropriate medical triage. Developed as an emergent response to the SARS outbreak in Taiwan, these interventions were rapidly implemented, leaving little time available to develop hotline data-collection instruments, train hotline staff, or prospectively plan for intervention evaluation. Despite these challenges, the interventions were evaluated by using available data, and a rapidly implemented population-based survey of Taipei city residents.

Approximately 50% of calls to the population-wide fever hotline did not result in referrals for further evaluation of fever, suggesting they were complaints unrelated to fever. In Taipei, 37% of respondents with body temperature recorded had fevers, a low proportion for a hotline intended for persons with fever. The low proportion of febrile persons is likely partly due to the definition of fever used in this evaluation. These results might also be partially due to worried-well callers. To improve appropriate use of a dedicated SARS fever hotline, media messages should be refined and the use of alternative resources for answering more general questions about SARS should be encouraged. During the outbreak, the Center for Disease Control of Taiwan established a public information line about SARS. If a fever hotline is used in future outbreaks, callers could be referred to the public information line with questions about temperature measurement, travel concerns, and other issues not directly related to a current febrile illness. The dedicated hotline could then focus on addressing its stated objectives more efficiently.

In the population-based survey, almost all respondents knew about the body-temperature monitoring campaign, and 71% knew about the fever hotline. The Bureau of National Health Insurance was highly successful in publicizing the campaign and hotline and should consider using similar methods for future hotlines.

An important aspect of this evaluation is assessing the potential impact of these interventions on improving early SARS detection. Eighteen callers to the fever hotline were identified as being at high risk for SARS. Because these persons were not followed up for outcome, determining if any subsequently met the World Health Organization’s suspected or probable SARS case definition was not possible. Furthermore, because hotline data were not always collected systematically, determining if all callers at high risk for SARS were identified was difficult. Lastly, sparse risk factor data limit our ability to determine if more persons at high risk for SARS should have been identified. Taking these limitations into account, the hotline potentially identified an estimated cohort of persons at high risk for SARS equivalent to 9.5% of the 190 suspected and probable SARS cases reported in Taiwan in the same 10-day period.

Documentation of the advice and referrals given by physicians was missing for a substantial proportion of calls; therefore, judging whether these callers were appropriately referred is not possible. The reasons for missing data are not yet fully elucidated. A telephone survey of callers to the Taipei SARS fever hotline is in progress to assess advice and referrals given and caller compliance. An algorithm and accompanying questionnaire that includes clearly articulated steps to measure temperature and document risk factors might assist in standardizing risk assessment, advice, referrals, and evaluation in future outbreaks (See Appendix).

## Supplementary Material

Appendix Description of Population-wide Body-Temperature Monitoring Campaign and SARS Fever Hotline
