# Supplementary material for: Body Temperature Monitoring and SARS Fever Hotline, Taiwan
Source: Emerg Infect Dis. 2004 Feb;10(2):373–6. doi: 10.3201/eid1002.030748 (PMC3322900; doi:10.3201/eid1002.030748)
Supplement: Appendix — Description of Population-wide Body-Temperature Monitoring Campaign and SARS Fever Hotline [file 03-0748-app-s1.pdf]

Appendix

Description of Population-wide Body-Temperature Monitoring Campaign and SARS Fever Hotline

In Taiwan, a National Temperature Monitoring Campaign and SARS fever hotline were implemented during June 1 to 10, 2003. This novel intervention was aimed at raising public awareness about SARS (and fever as one of its early signs), improving early detection of possible SARS cases, and preventing SARS transmission in healthcare facilities and the community through appropriate triage and medical referral. As part of this initiative, individual hospitals were required to monitor the temperatures of all healthcare workers and patients and to report to the Department of Health the number of persons monitored and number of fevers daily. All community residents were also asked to measure their body temperatures twice daily. Procedures for correctly measuring temperature and forms for recording temperatures were available to the public on a special SARS Web site and in pamphlets at gas stations and convenience stores. Tympanic thermometers and thermometer covers were provided to each village and prefecture in Taiwan. The body-temperature monitoring campaign lasted 10 days, June 1–10, 2003, and the fever hotline was available for several weeks.

Taiwan SARS Fever Hotline, Taipei

Appropriate triage by using the algorithm (Figure 3) and the number of possible SARS cases identified were evaluated by using data from the Taiwan SARS fever hotline.

Cross-Sectional Telephone Survey of Taipei Residents

Knowledge of the body-temperature monitoring campaign and fever hotline, compliance with the body-temperature monitoring campaign as defined by twice-daily temperature monitoring, and use of the fever hotline in the event of a fever were evaluated by using data from the cross-sectional telephone survey. To include those households not in an electronic version of the telephone listings, the last 2 digits of each selected number were replaced with 2 randomly selected digits. Interviewers used a script to outline the survey, explain that participation was voluntary and confidential, and obtain verbal consent. Names of participants were not recorded.

The brief telephone survey questionnaire included household demographics, knowledge of and compliance with the body-temperature taking campaign, sources of information about the campaign, occurrence of fever in the household during June 1 to 10, 2003, use of the fever hotline, and compliance with the hotline physician's advice. Inclusion criteria were residence in Taipei with a home telephone, and age ≥20 years. If more than one person at a telephone number met the eligibility criteria and was at the residence at the time of the call, one person was selected by convenience sampling to be interviewed (Table 3).
